# Nutritional Intervention for Sjögren Disease: A Systematic Review

**DOI:** 10.3390/nu17172743

**Published:** 2025-08-25

**Authors:** Fernanda Luiza Araújo de Lima Castro, Joyce Elisa Heredia, Lauren Frenzel Schuch, José Alcides Almeida de Arruda, Maurício Augusto Aquino Castro, Débora Cerqueira Calderaro, Marina Chaves de Oliveira, Sílvia Ferreira de Sousa, Tarcília Aparecida Silva

**Affiliations:** 1 Department of Oral Surgery, Pathology, and Clinical Dentistry, School of Dentistry, Universidade Federal de Minas Gerais, Av. Pres. Antônio Carlos, 6627, Room 3204, Belo Horizonte 31270-910, Brazil; fernandaluiza.alc@gmail.com (F.L.A.d.L.C.); alcides_almeida@hotmail.com (J.A.A.d.A.); mauricioaacastro@gmail.com (M.A.A.C.); silviafsousa@yahoo.com.br (S.F.d.S.); 2 Immunometabolism, Department of Nutrition, School of Nursing, Universidade Federal de Minas Gerais, Belo Horizonte 31270-910, Brazil; joyceheredia.jh@gmail.com (J.E.H.); marina.cdoliveira@gmail.com (M.C.d.O.); 3 Molecular Pathology Area, School of Dentistry, Universidad de la República (UDELAR), Montevideo 14600, Uruguay; laurenfrenzel@gmail.com; 4 Department of Oral Diagnosis and Pathology, School of Dentistry, Universidade Federal do Rio de Janeiro, Rio de Janeiro 21941-902, Brazil; 5 Departament of Locomotor System, School of Medicine, Universidade Federal de Minas Gerais, Belo Horizonte 31270-910, Brazil; dccalderaro@gmail.com

**Keywords:** diet, food, nutrition, Sjögren disease, Sjögren’s syndrome, xerostomia

## Abstract

**Background/Objectives:** The impact of nutritional interventions on Sjögren disease (SD) remains uncertain, and no standardized guidelines currently exist for managing its *sicca* symptoms. This systematic review evaluated the effects of dietary interventions on the symptoms of dry mouth and dry eyes in individuals with SD. **Methods:** Electronic searches were performed in four databases, supplemented by manual searches and searches of the gray literature. Both human and animal studies were included. The methodological quality of the selected studies was appraised, and the data were analyzed descriptively. **Results:** A total of nineteen studies (ten in humans and nine in animal models) were included. The treatments evaluated were dietary supplements, vitamins, medicinal herbs, and specially modified diets. The primary outcomes assessed included unstimulated and stimulated whole salivary flow rates, salivary-gland inflammation, and ocular dryness (Schirmer test). In animal models of SD, interventions such as caloric restriction, gluten-free diets, low-fat diets, and supplements (e.g., resveratrol, triptolide, and *Lycium barbarum* polysaccharide) were associated with increased salivary flow and reduced glandular inflammation. Conversely, diets rich in saturated fats were associated with reduced salivary flow and increased lymphocytic infiltration in salivary glands. Human studies yielded mixed results, with some reporting improvements in salivation following interventions with vitamins, herbal supplements, gluten-free diets, liquid diets, and whole-food, plant-based diets. **Conclusions:** Although dietary management may alleviate *sicca* symptoms and improve nutritional status in SD, the current evidence is insufficient to support specific recommendations for the management of oral symptoms.

## 1. Introduction

Sjögren disease (SD) is an autoimmune condition that primarily affects the salivary and lacrimal glands, leading to inflammation and resulting in symptoms such as dry mouth and dry eyes [1]. Its pathogenesis reflects a multifactorial process involving genetic, environmental, and hormonal factors that collectively drive a lymphocyte-mediated immune response [2,3,4]. Three key pathological events characterize SD: (i) an initial triggering phase influenced by genetic susceptibility, hormonal modulation, and environmental exposures; (ii) epithelial cell dysregulation within the salivary glands; and (iii) chronic inflammation marked by lymphocytic infiltration, B-cell hyperactivity, and the production of anti-SSA/Ro and anti-SSB/La autoantibodies. These mechanisms contribute to sustained tissue damage and potential systemic involvement [2].

SD predominantly affects middle-aged women [5]. Although not typically life-threatening, SD has no cure and may lead to systemic complications involving the pulmonary, renal, and vascular systems, as well as an increased risk of lymphoma [1,6]. Additionally, SD frequently coexists with other autoimmune diseases, such as rheumatoid arthritis (RA) and systemic lupus erythematosus (SLE) [3,5]. Treatment primarily focuses on symptom management, including salivary substitutes, stimulants, and artificial tears for *sicca* symptoms. In cases of systemic involvement, therapeutic strategies involve immunosuppressive and immunomodulatory agents, such as corticosteroids, methotrexate, mycophenolate mofetil, and biologics targeting B cells [7,8].

The reduction in salivary flow due to glandular damage is a common complaint that significantly impacts quality of life and oral health, increasing the risk of dental caries, fungal infections, and difficulties in eating [9,10,11]. Patients with SD may experience altered or diminished perception of smell and taste, challenges with chewing and swallowing, and dehydration of food, further impairing nutrient intake and dietary habits [12,13]. The role of diet in managing autoimmune diseases has been widely reported [14,15], including in SD [16,17,18,19,20]. However, studies on nutritional interventions to alleviate dryness-related symptoms remain scarce [13,16]. Consequently, there are currently no established consensus guidelines or protocols to effectively relieve symptoms and improve the nutritional well-being of individuals with SD.

The purpose of the present study was to synthesize the available data on nutritional interventions targeting clinical features, oral and ocular symptoms, and overall outcomes in individuals with SD. These findings may aid multidisciplinary teams in providing more comprehensive healthcare for patients with SD.

## 2. Methods

### 2.1. Eligibility Criteria

Articles examining nutritional interventions and their outcomes related to oral and ocular dryness in SD were included. The group of eligible study designs comprised case reports, case series, randomized controlled trials (RCT), and experimental studies using animal models. The search was restricted to English-language publications, excluding narrative reviews and inaccessible articles.

### 2.2. Databases and Search Strategy

Searches were conducted in PubMed, Embase, Web of Science, and Scopus in September 2023 and updated in July 2025. Search terms were combined using the Boolean operators “OR”, “AND”, and “NOT”. No restrictions were applied regarding the publication period. The full search strategy is detailed in Supplementary File S1. Duplicate references identified across databases were removed using Endnote (Clarivate Analytics, Toronto, ON, Canada). Additionally, a manual search was performed by cross-checking reference lists. The gray literature was also assessed, using Google Scholar, with the search limited to the first 200 records [21].

### 2.3. Selection Process and Data Extraction

Two authors (F.L.A.L.C. and J.E.H.) independently and blindly screened titles and abstracts. Full-text articles meeting the eligibility criteria were included. If the title or abstract was unclear, the full article was reviewed to determine inclusion or exclusion. Discrepancies were resolved by consensus or, if necessary, in consultation with a third author (L.F.S.).

Data were independently extracted by two authors (F.L.A.L.C. and J.E.H.) using a standardized form. For human studies, the following information was collected: authors’ names, year of publication, country, sample size, clinical features of SD, nutritional intervention, duration of intervention, and outcomes related to dryness symptoms. For animal studies, data were gathered on the specific animal models used (e.g., strain, age, and sex), the nutritional intervention applied, and the histological evaluation of salivary glands to assess the outcomes related to SD.

### 2.4. Quality Assessment

Two authors (F.L.A.L.C. and J.E.H.) independently assessed the methodological quality of the studies. The Joanna Briggs Institute (JBI) critical appraisal tools were used for clinical studies, including case reports [22], case-control studies [22], and randomized controlled trials [23]. Animal studies were evaluated using the SYRCLE Risk of Bias tool [24]. The assessment focused on key aspects such as study design, risk of bias, confounding variables, sample size, outcome measurements, and result reproducibility. Studies were evaluated according to the specific parameters applicable to each study type. Any discrepancies between the two authors were resolved through a discussion with a third author (L.F.S.), which lasted until consensus was reached.

### 2.5. Data Analysis

The data were tabulated in Microsoft Office Excel 2019 (Microsoft^®^, Redmond, WA, USA) and analyzed descriptively.

### 2.6. Protocol and Registration

This systematic review was conducted following the guidelines of the Preferred Reporting Items for Systematic Reviews and Meta-Analyses (PRISMA) statement [25]. A protocol was drafted and registered with the National Institute for Health Research International Prospective Register of Systematic Reviews (PROSPERO; No. CRD42024517376).

## 3. Results

### 3.1. Study Selection and General Characteristics of Included Studies

**Figure 1:**
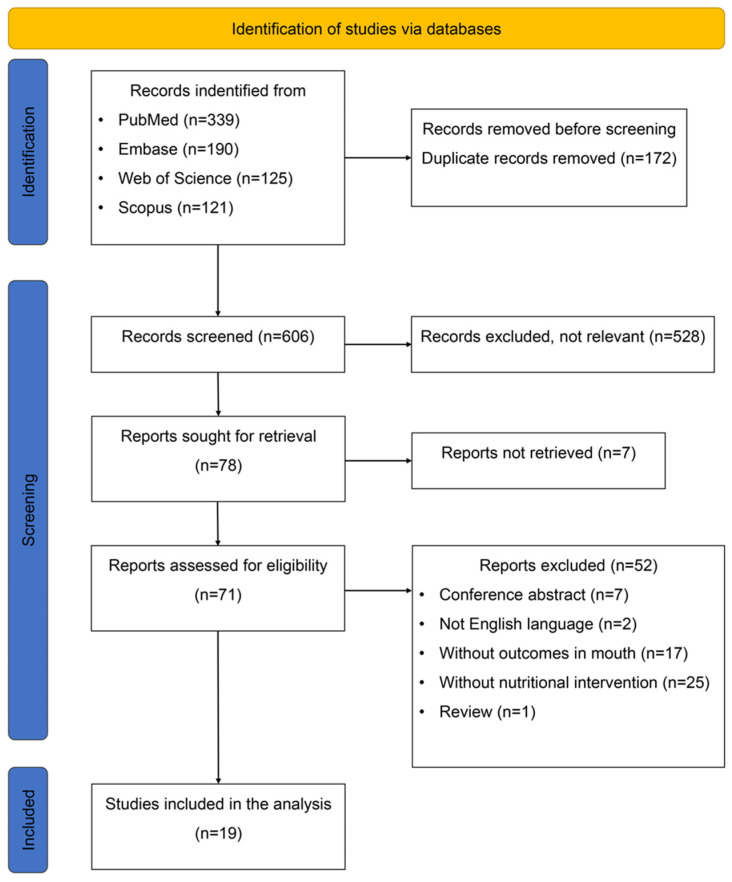
PRISMA Flowchart depicting article selection process.

The electronic searches initially identified 775 articles. After applying the eligibility criteria, 16 studies were selected. Additionally, three studies were identified through manual searches and included in the final analysis. Figure 1 depicts the flowchart outlining the article selection process. Summaries of the data extracted from the nine studies involving animal models and the ten human studies are displayed in Table 1 and Table 2, respectively.

### 3.2. Animal Studies

The animal studies were published between 1989 and 2024 and conducted in eight countries. To recapitulate the clinical features of SD, different mice lineages were used, including female hybrid New Zealand mice (NZB × NZW) [26,27], non-obese diabetic (NOD) mice (or mouse) [28,29,30,31,32,33], and male IL-14α transgenic mice [20]. Nutritional interventions were administered either orally or via gavage. Studies have evaluated the effects of resveratrol, triptolide, and *Lycium barbarum* polysaccharide (LBP), as well as dietary modifications, including high-saturated- or high-unsaturated-fat diets, gluten-free (GF) diets, alternate-day fasting (ADF), and caloric restriction (CR). The durations of these dietary interventions ranged from 3 weeks to 11 months.

The primary outcomes assessed in most studies included unstimulated salivary flow, the degree of salivary-gland inflammation as determined through histopathological analysis, and the Schirmer test. Zhang et al. [20] evaluated the effects of a diet containing 60 kcal% fat over an 11-month period on the relevant parameters. Swanson et al. [26] compared the impact of a high-saturated-fat diet, a high-unsaturated-fat diet, and a low-fat diet over a 3-month period. Animals fed high-fat diets exhibited increased infiltration of inflammatory cells and structural damage to the salivary and lacrimal glands [20,26]. In contrast, those receiving the low-fat diet showed reduced lymphocytic infiltration and better preservation of glandular architecture. Tear secretion, assessed using the Schirmer test, was significantly higher in the low-fat group compared to both high-fat groups, indicating superior lacrimal gland function [20,26]. Conversely, CR strategies, such as ADF [32,33] and diets with a 40% calorie reduction [27], demonstrated positive effects, improving salivation and reducing salivary-gland inflammation. A GF diet, in which gluten-containing ingredients were replaced with meat protein for 13 weeks, resulted in reduced salivary-gland inflammation [30].

**Figure 2:**
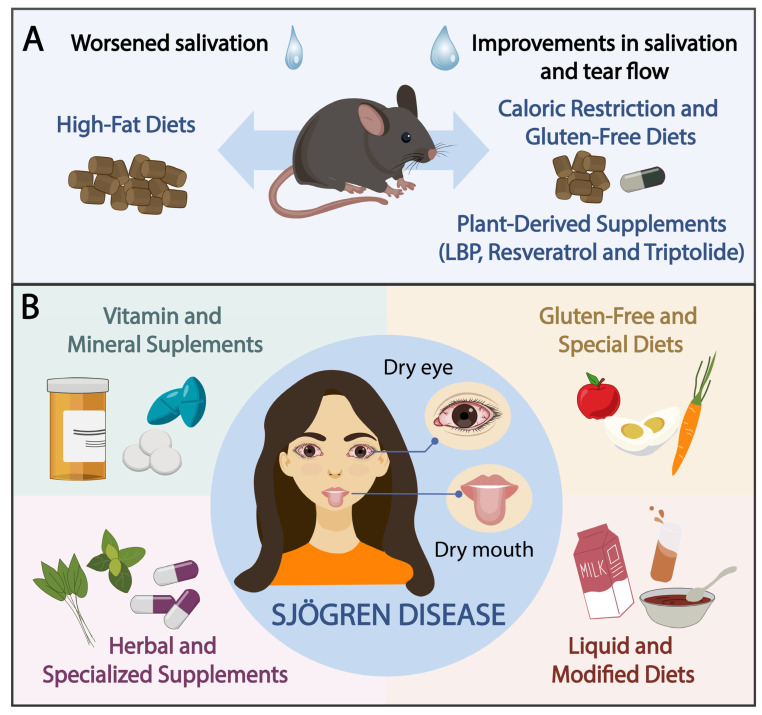
(**A**) Schematic representation of dietary interventions in mice and their effects on salivation and tear flow. High-fat diets were associated with worsened salivation, whereas caloric restriction, gluten-free diets, and plant-derived supplements (e.g., *Lycium barbarum* polysaccharide [LBP], resveratrol, and triptolide) improved both salivation and tear flow. (**B**) Summary of nutritional interventions in humans with Sjögren disease, focusing on dry mouth and dry eyes. Liquid diets significantly enhanced both salivary and tear production. The effects of vitamins and minerals were variable, with only one study reporting improvements in both salivation and tear flow. Gluten-free and specialized diets improved tear flow but showed no impact on dry mouth. Herbal and specialized supplements demonstrated promising effects on salivary flow but yielded inconsistent results for tear production.

Plant-derived supplements, such as resveratrol, triptolide, and LBP, demonstrated beneficial effects in animal models of SD compared to controls [28,29,31]. Resveratrol and triptolide were administered via gastric gavage for 16 weeks and 90 days, respectively, and both were compared to a control group. LBP was orally administered over a 12-week period, with one group receiving a high dose and another a low dose. These groups were compared to a control group and a group receiving low-dose recombinant human IL-2. Figure 2A summarizes the data obtained from these animal studies.

### 3.3. Human Studies

#### 3.3.1. Vitamin and Mineral Supplements

Raffle [34] investigated the effects of vitamin A, ferrous sulfate, and Beplex capsules (containing thiamine hydrochloride, riboflavin, and nicotinic acid) in a patient with SD symptoms. The study did not include objective measurements of salivary or tear flow, but reported no improvement in oral health conditions, despite the patient’s subjective perception of improvement.

Horrobin and Campbell [36] evaluated the effects of vitamin C, pyridoxine, and Evening Primrose Oil capsules on SD patients. This 12-month intervention significantly improved tear and saliva production in five individuals. However, McKendry [37] reported that the same nutrients were ineffective in managing oral and ocular dryness symptoms in SD.

Liao et al. [43] investigated the effects of potassium citrate and vitamin D, reporting that the patient experienced no *sicca* symptoms following treatment. However, the study did not include objective measurements to substantiate these reported improvements.

#### 3.3.2. GF and Special Diets

Maclaurin et al. [35] documented the effects of a GF diet combined with vitamin B12, folic acid, calcium, and other vitamins over a two-month period. This regimen improved tear secretion, as measured by the Schirmer test, and overall well-being; however, the dry mouth symptoms remained unchanged.

Goldner and Staffier [44] evaluated the effects of a whole-food, plant-based (WFPB) diet, consisting of raw foods, leafy greens, vegetables, water, and polyunsaturated fatty acids (PUFAs), over a four-week period. The study reported improvements in both dry mouth and dry eyes in three patients with SD. However, no objective tests for tear or salivary flow were conducted.

#### 3.3.3. Liquid and Modified Diets

Peen et al. [40] investigated the effects of a liquid diet that eliminated the need for chewing and found a significant increase in unstimulated whole salivary flow rate (UWSFR), which rose from 1.18 mL/15 min to 1.70 mL/15 min over four weeks. Similar improvements were observed in tear flow, as measured by the Schirmer test, while no such effects were noted in the control group.

#### 3.3.4. Herbal and Specialized Supplements

Pedersen et al. [38] investigated Longo Vital, a herbal-based vitamin supplement. The study showed a significant increase in UWSFR in one group after four months. Results regarding stimulated salivary flow were mixed, with improvements observed during different phases of treatment. No significant changes were observed in dry eye symptoms.

Singh et al. [42] evaluated the effects of TheraTears Nutrition, a supplement containing flaxseed oil, eicosapentaenoic acid, docosahexaenoic acid, and vitamin E, providing a total of 1750 mg of omega-3. The supplement significantly improved UWSFR and stimulated salivary flow; however, these improvements were not significantly different from those observed in the placebo group. Additionally, no significant changes were noted in Schirmer test results for either group. Another study conducted by Al-Rawi et al. [45] evaluated the effects of omega-3 supplementation over a two-month period in patients with SD and reported improvements in UWSFR, Schirmer test results, and symptoms of both oral and ocular dryness. Figure 2B summarizes the nutritional interventions conducted in humans.

### 3.4. Critical Appraisal

Critical appraisal of the case reports revealed that most articles provided a clear description of the patient’s demographic characteristics, medical history, and clinical timeline. Additionally, the majority adequately detailed the diagnostic tests or assessment methods used, as well as the interventions, treatment procedures, and post-intervention clinical outcomes. However, two articles did not include information on the patient’s contemporaneous clinical condition. Also, most reports failed to identify or describe adverse events or unexpected outcomes. Nevertheless, all articles presented key takeaway lessons. The case–control study lacked clarity in defining the case and control groups, assessing outcomes, and determining the exposure period. Furthermore, it did not outline strategies for managing confounding factors. Two randomized clinical trials raised concerns regarding participant randomization, treatment group allocation, blinding of assessors, outcome measurement, and statistical analysis. In contrast, a third trial was conducted with greater methodological rigor and did not present major deficiencies. Supplementary Files S2–S4 provide details on the critical appraisal of human studies. The quality assessment of animal studies identified multiple articles with unclear information on various evaluatory criteria (Supplementary File S5).

## 4. Discussion

SD is a chronic systemic autoimmune disease characterized by severe dry mouth, which impairs chewing, swallowing, and overall nutritional status [16,46]. While evidence on the role of diet in SD management remains limited, it is well established that nutritional status influences the progression of rheumatic and inflammatory diseases [47,48]. Healthy dietary patterns can regulate gut microbiota, strengthen barriers against harmful antigens, reduce chronic inflammation, and alleviate autoimmune disease symptoms [49]. This systematic review analyzed both observational and experimental studies in humans and animal models, focusing on nutritional interventions for SD. Various dietary components, including plant-derived compounds, vitamins, omega-3 fatty acids, and specific dietary patterns, demonstrated varying degrees of efficacy in alleviating oral symptoms and reducing salivary-gland inflammation. However, the heterogeneity of study designs and outcome measures, along with the limited number of high-quality human studies, restricts the ability to derive specific nutritional recommendations.

Plant-derived compounds such as resveratrol and triptolide have been extensively studied for their immunomodulatory properties. Resveratrol regulates T-cell differentiation and inhibits pro-inflammatory cytokines through the AMPK and NF-κB pathways [50], while triptolide modulates NF-κB, MAPK, IL-6/STAT3, and SOCS3 signaling pathways [51,52]. Animal studies have demonstrated the therapeutic potential of these compounds, showing improvements in hyposalivation and reductions in salivary-gland inflammation. Additionally, resveratrol increased IL-10 expression, whereas triptolide inhibited JAK/STAT and NF-κB signaling [28,29]. Nevertheless, neither compound has been tested for its effects on dry eye symptoms. LBP, extracted from the *goji berry*, has also exhibited antioxidant and anti-inflammatory properties, improving dry mouth symptoms in animal models [31,53]. However, no studies have evaluated its impact on dry eye symptoms.

Recent evidence suggests that dysregulation of lipid metabolism plays a critical role in autoimmune diseases, including SD. Patients with SD frequently exhibit dyslipidemia, characterized by elevated levels of total cholesterol, LDL, and triglycerides, along with reduced HDL levels [54]. These lipid imbalances have been associated with increased systemic inflammation and glandular dysfunction. In the present review, we observed that nutritional interventions, such as caloric restriction and plant-derived compounds, demonstrated promising effects in reducing glandular inflammation. These findings may be partially attributed to their role in modulating lipid metabolism, as excessive lipid deposition in exocrine tissues has been proposed as not merely as a consequence of the disease, but a potential trigger for inflammation [54].

Vitamins play a crucial role in immune regulation and cellular metabolism, with deficiencies linked to the development of autoimmune diseases [55]. Vitamins A, D, and E influence various aspects of both the innate and adaptive immune systems, contributing to cellular regulation and metabolism. Deficiencies in vitamins A, D, and E have been implicated in the pathogenesis of autoimmune disorders [50,55,56]. Szodoray et al. [17] investigated vitamin A, D, and E levels in patients with SD, reporting significantly lower vitamin A levels in individuals with more advanced disease compared to those with milder forms. In this context, reduced vitamin A levels may contribute to disease progression, as patients with lower serum levels exhibited more severe symptoms. Vitamin C also plays a role in immune function and autoimmunity [57]; however, evidence regarding its benefits in managing SD and *sicca* symptoms remains inconclusive. Notably, one of the vitamin-based supplements evaluated (Longo Vital) was associated with adverse effects, including constipation, pruritus, skin rash, nausea, and exacerbation of psoriasis [38].

Omega-3 polyunsaturated fatty acids have demonstrated anti-inflammatory effects in autoimmune diseases by reducing oxidative stress and modulating pro-inflammatory cytokines [58,59]. Patients with SD, in particular, may have insufficient omega-3 intake, which has been associated with worsening disease symptoms, including reduced salivary flow [60]. A recent literature review [61] evaluating the use of PUFAs, including omega-3, indicated potential benefits for keratoconjunctivitis and for the modulation of inflammatory markers, such as total plasma phospholipid levels, erythrocyte membrane fatty acid composition, and ocular surface inflammation. Supporting these observations, Al-Rawi et al. [45] reported improvements in both salivation and tear flow following two months of omega-3 supplementation. Additionally, Singh et al. [42] documented that omega-3 supplementation increased salivary flow and improved periodontitis by reducing probing depth in patients with SD. From a clinical perspective, while a previous systematic review found no evidence of an increased risk of periodontitis in SD patients, it highlighted that these patients are more prone to dental caries, compared to individuals without SD [62]. Mechanistically, periodontitis may still be linked to SD through shared inflammatory pathways and dysregulated immune responses. This suggests that improving periodontal health could potentially alleviate other systemic symptoms of the disease.

Two studies, one in humans and one in mice, evaluated the effects of a GF diet on SD, but neither reported positive outcomes concerning oral health [30,35]. Gluten is a protein known to trigger adverse effects in individuals with celiac disease, and it has been implicated in exacerbating symptoms of autoimmune disorders [63,64]. A recent review found that GF diets alleviated symptoms in many patients with non-celiac autoimmune diseases, suggesting potential benefits for individuals with autoimmune conditions [65]. However, studies assessing this dietary approach in SD did not find improvements in oral symptoms, despite reporting other benefits, such as reduced pancreatic insulitis and decreased lung and spleen weight in mice, as well as enhanced general well-being and increased tear secretion, as confirmed by the Schirmer test, in humans [30,35].

The modern dietary pattern, particularly in Western countries, is characterized by the consumption of ultra-processed foods rich in sugar and fat [66]. Such diets are known to induce gut microbiota dysbiosis, which promotes inflammatory changes, enhances autoantibody production, and contributes to the development of autoimmune diseases [67,68]. Emerging evidence suggests a link between the gut microbiota and salivary-gland function, known as the gut–salivary-gland axis [69]. Dysbiosis is associated with systemic inflammation, potentially driving autoimmune mechanisms in SD [70]. Dietary interventions aimed at modulating the microbiota (e.g., probiotics and prebiotics) may help regulate autoimmune responses and alleviate dryness symptoms, warranting further investigation. Interestingly, one study found that patients with SD who also had metabolic syndrome, i.e., a cluster of cardiovascular risk factors influenced by nutrition, genetics, and gut microbiota [71], exhibited increased body mass index (BMI), waist circumference, visceral fat, hypertension, and diabetes, as well as elevated cholesterol levels [72]. Nevertheless, other studies did not demonstrate significantly higher BMI values or an increased prevalence of obesity in SD patients [73]. These inconsistencies, however, highlight the importance of tailoring dietary strategies to specific patient subgroups to enhance treatment efficacy and optimize clinical outcomes.

A diet rich in fruits, vegetables, legumes, fish, grains, and olive oil—commonly referred to as the Mediterranean or plant-based diet—has been shown to prevent or mitigate various chronic conditions [74,75]. Reducing the intake of processed and high-fat foods while adopting a balanced dietary pattern can alleviate inflammation and musculoskeletal pain in patients with RA and SLE [76,77]. Three of the studies included in the present review addressed such dietary influences. One reported that animals fed diets high in saturated or unsaturated fats exhibited more pronounced lymphocytic infiltration in the salivary glands compared to those on a low-fat diet [26]. Another found that high-fat diets reduced salivary secretion, increased inflammation, and worsened gut dysbiosis [20]. Although no human studies have directly assessed the impact of unbalanced diets on salivary-gland function, one plant-based diet intervention revealed symptomatic improvement, with some patients discontinuing medications; however, outcomes were based solely on self-reported data, without objective validation [44]. Recently, Chaaya et al. [78] provided human evidence linking higher adherence to the Mediterranean diet with reduced ocular dryness in patients with SD. Moreover, a higher intake of polyunsaturated fatty acids (e.g., omega-3s from fish, nuts, and olive oil) was independently associated with better ocular surface health. Collectively, these findings suggest that unbalanced diets may exacerbate both salivary and ocular symptoms in SD. Although preclinical data support this association [19,73,77], the inclusion of recent clinical evidence reinforces the therapeutic potential of Mediterranean-style dietary strategies.

Another type of intervention analyzed in this review was based on ADF and CR. Mice subjected to ADF exhibited significantly increased salivary flow and markedly reduced inflammation in the submandibular glands [32]. Additionally, ADF has been shown to decrease the expression of markers related to senescence, apoptosis, and inflammation in salivary-gland stem cells [34]. These markers are associated with various disorders, including autoimmune diseases such as SD [79,80,81]. Chandrasekar et al. [27] conducted a longitudinal study in which animals were evaluated from a young age through advanced stages, and compared an ad libitum diet with a 40% caloric restriction regimen. The authors found that early initiation of caloric restriction prevented inflammation in the salivary glands. However, these two studies did not assess salivary flow [27,33]. Hunger is believed to modulate immune responses and alleviate symptoms of autoimmune diseases. Food deprivation, as a dietary intervention, has been associated with improved glycemic control, delayed aging, suppression of inflammation, and enhanced overall health [82,83]. Studies investigating caloric restriction in animal models of multiple sclerosis have demonstrated a downregulation of pro-inflammatory genes, leading to significant symptom improvement [84]. Further research is required to assess the safety of this approach and establish an optimal food deprivation regimen.

Peen et al. [40] investigated the effects of a liquid diet, which eliminates chewing, in individuals with SD. Notably, this dietary approach was found to be beneficial, increasing salivary flow after just four days. The study was based on the hypothesis proposed by Humphreys-Beher et al. [85], who suggested that salivary-gland stress could be mitigated by reducing neural input to the glands, and that this could be achieved through decreased mastication. However, these findings contrast with those of other studies [86,87]. A recent literature review compiled multiple studies suggesting that soft diets tend to have a negative impact on the salivary glands [88]. The parotid glands were the most affected, exhibiting atrophy, reduced weight, and decreased production of amylase and salivary proteins, whereas the submandibular and sublingual glands remained largely unaffected [86,87]. Given the limited number of studies assessing diet consistency and its effects on SD, it remains inconclusive whether a liquid diet provides a genuine benefit for patients with dry mouth.

Nutritional alterations in SD should be considered in light of their impact on the oral microbiome, which may contribute to immune dysregulation and oral dryness. It has been documented that oral dysbiosis is increasingly recognized as central to SD pathophysiology [89,90]. For instance, *Prevotella melaninogenica* has been associated with pro-inflammatory responses through MHC, CD80, and IFN-γ induction [91], whereas *Haemophilus parainfluenzae* appears to be protective; i.e., its reduced salivary levels in SD contrast with murine data showing that glandular inoculation restored salivary function, reduced sialoadenitis, and suppressed IFN-γ–producing T cells, with epithelial exposure inhibiting human CD8+ T-cell activation [92]. Despite evidence linking dysbiosis to symptom exacerbation, the influence of diet on the oral microbiome in SD remains poorly explored, though dietary composition directly shapes microbial substrates and the production of pro- or anti-inflammatory metabolites [93], offering potential insights into mechanisms of chronic inflammation and disease progression.

As far as we know, this is the first systematic review to comprehensively synthesize information on the role of nutrition in the dryness-related aspects of SD, drawing from both animal models and human studies. A key strength of this review lies in its rigorous, protocol-registered methodology. The inclusion of animal studies was intentional and justified by their capacity to elucidate mechanistic and immunometabolic pathways that remain unexplored in human cohorts. Although preclinical findings are not directly generalizable, they offer critical translational insights in a field where clinical trials remain scarce. Conversely, limitations of the present systematic review should be acknowledged. First, many of the included studies lacked robust methodological details and comprehensive data reporting, which limited the depth of evidence synthesis. Second, there was marked heterogeneity in study designs, including variability in population characteristics, types and durations of nutritional interventions, and outcome measures (e.g., unstimulated vs. stimulated salivary flow, and differing Schirmer test methodologies). This variability hinders direct comparisons across studies, and the feasibility of meta-analyses. Third, evolving classification criteria for SD in recent years may have influenced patient selection and stratification across studies. Additionally, the absence of standardized tools for assessing dryness parameters further limits the interpretability of results. Future randomized clinical trials and well-designed cohort studies are essential to advance this field. Such studies should adopt current diagnostic guidelines [86], incorporate parameterized assessments of oral dryness, and apply standardized nutritional interventions [94]. Lastly, while animal models effectively recapitulate key aspects of SD pathophysiology and are valuable for exploring the effects of dietary modulation, the extrapolation of these findings to human disease remains limited due to inherent differences in immune regulation, disease progression, and environmental context.

In summary, several present dietary approaches are promising for SD management. Based on the available evidence, the following nutritional strategies may be considered for recommendation:

- High-fat diets (saturated and unsaturated) should be avoided, as they are linked to increased inflammatory infiltration, glandular damage, and inferior Schirmer performance [20,26].
- CR strategies, including alternate-day fasting and ~40% energy reduction, have shown benefits with respect to salivation and inflammation in animal studies [27,32,33].
- GF diets have reduced salivary-gland inflammation in animals and improved Schirmer without change in xerostomia in humans [30,35].
- WFPB [44] and liquid/modified diets that eliminate mastication are linked with symptom improvement [40].
- Plant-derived supplements such as resveratrol, triptolide, and *Lycium barbarum* polysaccharide have demonstrated benefits in animal models [28,29,31].

Evidence for vitamins/minerals and other specialized supplements (e.g., vitamin A, iron, Beplex; vitamin C, pyridoxine; potassium citrate with vitamin D; Longo Vital; and omega-3 formulations) remains mixed or limited, with variable results, and one trial showing no advantage over placebo [34,36,37,38,42,43,45]. Further research is needed to establish a clear and specific dietary recommendation for patients with SD.

The present review does not merely reaffirm the broad notion that healthy nutrition contributes to immune homeostasis. Rather, it offers a focused, disease-specific synthesis of dietary strategies that have been tested in the context of SD, particularly in relation to its hallmark features, i.e., exocrine gland dysfunction, salivary hypofunction, and chronic lymphocytic infiltration. By integrating findings from experimental models and clinical studies, we emphasize the role of nutritional interventions (e.g., caloric restriction, omega-3 supplementation, plant-derived immunomodulators, and gut-microbiota-targeting strategies) not as general wellness measures, but as potential modulators of key immunometabolic pathways involved in SD pathogenesis. This conceptual distinction is essential to prevent overgeneralization and to position nutrition as a translational tool in the management of SD, beyond its ancillary role in systemic inflammation.

## 5. Conclusions

Dietary management may be a viable strategy for alleviating *sicca* symptoms and improving the nutritional status of individuals with SD. Early studies, including those on GF diets and vitamin supplementation, have yielded mixed results, with no definitive improvements in dry mouth symptoms. Similarly, the effects of individual nutrients and dietary patterns, such as CR and plant-derived compounds, require further investigation. More recent research on WFPB diets, liquid diets, and specific supplements suggests potential benefits. Overall, this systematic review underscores the need for well-designed clinical trials and standardized assessment methods to better understand and optimize nutritional strategies for patients with SD.

## Figures and Tables

**Table 1:** Summary of animal studies retrieved in the systematic review.

Study	Country	Sample	Animal Model	Sex	Age	Disease Features	Nutritional Intervention	Intervention Duration	Stimulated Salivary Flow Rate	Histological Analysis	Schirmer Test
Swanson et al., 1989 [26]	USA	60 cases and 24 controls	Weaned NZB × NZW mice	F	3- to 4-week-old	The NZB/W mice also developed autoimmune-related exocrine gland disease, resembling the abnormalities observed in SD	Diets: (a) 20 animals: HSF (7.7% coconut oil, 1–3% corn oil); (b) 20 animals: HUF (9% corn oil); (c) 20 animals: LF (1.2% corn oil); Control group: 24 animals fed a conventional rodent diet.	3 months	NI	Destructive infiltration of exocrine glands: HSF: 47%; HUF: 41%; LF: 23%; Control group: 25%.	HSF: 2.2 mm (0.73) HUF: 2.2 mm (0.97) LF 3.3 mm (0.78) Control group: 3.0 mm (1.6)
Chandrasekar et al., 1995 [27]	USA	NI	(NZB × NZW) F1	F	4-week-old	Mice developed age-associated sialoadenitis, characterized by the infiltration of inflammatory mononuclear cells in the salivary glands, resembling human SD	Mice were fed a semi-purified diet containing either 5% fat AL or 40% CR. The diet composition included the following ingredients: casein (20%), corn oil (5%), starch (32%), dextrose (33%), fiber (4.5%), DL-methionine (0.3%), choline chloride (0.2%), salt mixture (3.5%), and a vitamin mix (vitamin diet fortification) (1.5%). To compensate for decreased food intake, the CR diet was supplemented with twice the amount of the vitamin and mineral mixture. CR mice were initially fed 5–10% less than the AL group for the first two weeks, 10–20% less during the third and fourth weeks, and 40% less thereafter.	3.5 (young) to 8.5 (old) months	NI	Young animals: AL and CR—presented normal salivary-gland tissues. Older animals: CR—patchy foci of mild chronic peri-acinar and periductal inflammation of 0.247 ± 0.120 (*n* = 12) and percentage area of inflammation 0.130 ± 0.067%; AL—dense confluent infiltrates of lymphocytes, with average focus score of 0.824 ± 0.152 and percentage area of inflammation 2.410 ± 0.793% (*n* = 12; *p* < 0.005, comparing AL and CR mice).	NI
Inoue et al., 2016 [28]	Japan	NI	NOD/shi mice	F	6-week-old	NOD mice, in which loss of lacrimal and salivary-gland function occurred	The mice were orally administered the vehicle (Milli-Q) or resveratrol (Sigma-Aldrich, St. Louis, MO, USA) at doses of 100 or 250 mg/kg using gastric intubation 6 days per week from 6 to 20 weeks of age (*n* = 6 mice/group). Resveratrol was dissolved in 0.2 mL of H_2_O for administration.	16 weeks	The salivary flow rate was not altered by the resveratrol between 6 weeks and 20 weeks of age, whereas 250 mg/kg resveratrol showed protective effects on the hyposalivation observed in the NOD mice at age 22 weeks.	In the parotid and sublingual salivary glands, no lymphocyte infiltration was observed in any of the groups of NOD mice. The periductal inflammatory cell foci in submandibular glands were not affected by resveratrol administration.	NI
Guo et al., 2021 [29]	China	48	NOD mice	F	8-week-old	The authors used NOD mice, as an animal model of SD	The NOD mice were administered triptolide by means of gastric gavage. The dosage selection for TP [10 µg/(kg/day), 20 µg/(kg/day), and 40 µg/(kg/day)] was determined based on a previous study. Forty-eight NOD mice were divided into 4 groups: Control group (vehicle) (*n* = 12), NOD mice treated with 10 µg/(kg/day) TP (*n* = 12), NOD mice treated with 20 µg/(kg/day) TP (*n* = 12), and NOD mice treated with 40 mg/(kg/day) TP (*n* = 12)	90 days	The salivary flow rate of the control group decreased over time. With the increase in TP concentration, the difference between the TP group and the control group was larger.	Focus Score: Control = 3.1 ± 0.21; Triptolide 10 = 2.4 ± 0.18; Triptolide 20 = 1.8 ± 0.17; Triptolide 40 = 1.5 ± 0.19.	NI
Haupt-Jorgensen et al., 2022 [30]	Denmark	NI	NOD/BomTac mice	F	3- to 13-week-old	NOD mice are a widely used model for T1D (type 1 diabetes and SD)	Breeding pairs of prediabetic NOD mice were fed a gluten-free modified Altromin diet (the GF diet was prepared by replacing the gluten-containing ingredients with meat protein) or a standard non-purified Altromin diet (STD).	13 weeks	NI	Focus score: GF (score 0.7) and STD (score 3.8) diets (*p* = 0.124). Submandibular glands from GF versus STD mice: 47% (*p* = 0.042) fewer CD68+ cells and 49% (*p* = 0.037) fewer CD4+ cells. In the same organ, there were tendencies (*p* = 0.130–0.650) to demonstrating fewer CD20+ cells, more CD8+ cells and fewer VEGFR1+ and VEGFR2+ cells (NS).	NI
Wang et al., 2021 [31]	China	NI	NOD mice	F	7-week-old	A NOD mouse model was used, as it spontaneously develops features resembling human SD, such as lymphocytic infiltration in the salivary glands, hyposalivation, and autoantibodies	Use of *Lycium barbarum* polysaccharide (LBP) by oral administration. Mice were randomized into four groups (eight per group): Low-dose LBP (LBP.L) (5 mg kg^−1^ d^−1^), High-dose LBP (LBP.H) (10 mg kg^−1^ d^−1^), Low-dose recombinant human IL-2 (LDIL-2, 25,000 IU/d), and Control (saline water).	12 weeks	NOD mice treated with LBP had increased salivary flow rates compared with the control group.	LBP.L group (histology score: 1.88 ± 0.83, foci number: 0.92 ± 0.59). Control group (histology score: 3.38 ± 1.06, *p* = 0.014; foci number: 1.87 ± 0.92, *p* = 0.045; *n* = 8). LBP.H group (histology score: 2.38 ± 0.92, *p* = 0.206; foci number: 1.13 ± 0.53, *p* = 0.193). LDIL-2 treated group (histology score: 1.50 ± 0.76, *p* = 0.002; foci number: 0.50 ± 0.54, *p* = 0.002).	NI
Li et al., 2022 [32]	USA	NI	NOD mice	F	10-week-old	NOD mice, a well-defined mouse model that recapitulates human SD	The mice were deprived of food every other day from 10 to 13 weeks of age (ADF). All mice had unrestricted access to water throughout the entire experiment. Control: standard chow AL diet.	3 weeks	Mice in the ADF group exhibited higher salivary flow rate compared to the control group, suggesting the improvement of the salivary secretory function (*p* < 0.05).	H/E staining of submandibular gland sections showed significantly lower leukocyte focus numbers and infiltration areas in the mice with ADF than in the control mice (*p* < 0.05).	NI
Zhang et al., 2022 [20]	China	NI	WT and IL-14α transgenic mice (IL-14α TG)	M	NI	The authors used IL-14α transgenic mice (IL-14α TG), a mouse model that mimics the clinical features of SD in the same time frame as in humans. This mouse model not only shows lacrimal gland and salivary-gland inflammation but also shows systemic manifestations of the disease	Mice were fed a standard diet (SD, 10 kcal% fat, 1022) and a HFD (60 kcal% fat).	11 months	The levels of salivary-gland secretions of the IL14 HFD mice were significantly lower than the levels of salivary-gland secretions in the WT group and the IL14 group.	The results showed that the IL14 HFD group developed the same submandibular gland (SMG) injuries as the WT HFD group. However, the IL14 HFD group showed more extensive and severe lymphocytic inflammatory infiltration of SMG.	Tear production was significantly decreased in both the WT HFD and the IL14 HFD groups, compared with comparable SD groups
Li et al., 2024 [33]	Japan	NI	NOD mice	F	10-week-old	NOD mice with newly established SD	Mice were fed every other day (ADF) and age- and sex-matched mice were fed a standard chow AL diet (controls).	3 weeks	NI	The authors conducted immunohistochemical analysis: p16^INK4a^ expression in SMG were significantly reduced by ADF (*p* < 0.01); Expression levels of anti-apoptotic molecules BCL-2, BCL-XL, and MCL-1 in SMG were decreased by ADF; ADF decreased NLRP3 inflammasome expression level and, consequently, its products (IL-1β and IL-18).	NI

Note: ADF, alternate day fasting; AL, ad libitum; CR, calorie restriction; GF, gluten-free; HFD, high-fat diet; HSF, high in saturated fat; HUF, high in unsaturated fat; LF, low in fat; LBP, *Lycium barbarum* polysaccharide; NI, no information; NOD, non-obese diabetic; NS, not significant; NZB, New Zealand Black; NZW, New Zealand White; SD, Sjögren disease; STD, standard diet; TP, triptolide; USA, United States of America; WT, wild type.

**Table 2:** Summary of human studies retrieved in the systematic review.

Author and Year of Publication	Country	Sample	Sex	Age	Sjögren Disease Criteria	Additional Disease Information	Nutrition Intervention	Intervention Duration	UWSFR	Stimulated Salivary Flow Rate	Schirmer Test	Perceived Improvement in Dryness
Raffle, 1950 [34]	UK	1	F	50	NI	Symptoms: vague rheumatic pains, weakness, lack of appetite and energy, pronounced facial swelling at the parotid regions, swelling under the chin, dry mouth, dysphagia, soreness of the tongue.	First, vitamin A, 150,000 units daily. Then, vitamin A stopped, and ferrous sulfate 18 g daily started. Then, “Beplex” capsules, six per day, were given (6 mg of thiamin hydrochloride, 4.8 mg. of riboflavin, and 60 mg. of nicotinic acid).	NI	NI	NI	NI	No improvement on the buccal condition, but the patient subjectively felt better.
Maclaurin et al., 1972 [35]	New Zealand	1	F	73	NI	Dryness and soreness of the eyes and mouth for one year. Schirmer’s test confirmed diminished tear secretion.	GF diet and treatment with vitamin B12 by injection, folic acid, calcium, and other vitamins.	2 months	NI	NI	Improvement in tear secretion by Schirmer’s test	Dry mouth did not improve, improvement in general well-being.
Horrobin and Campbell, 1980 [36]	Canada	5	1 F 4 NI	52 4 NI	NI	One patient with a history of classic moderate rheumatoid arthritis and history of dry mouth and dry eyes, and the four others with long-established SD.	Intake of high-dose (7.5 g/day) vitamin C for a year, with 50 mg/day pyridoxine and 6 × 0.6 mL Evening Primrose Oil capsules per day.	12 months	NI	NI	NI	All five patients had substantial improvements in tear and saliva production.
McKendry, 1982 [37]	Canada	10	NI	NI	NI	Patients presenting with subjective oral and ocular dryness.	Vitamin C 3.0 g daily and Evening Primrose Oil 6 × 500 mgs. capsules daily for six weeks. Pyridoxine 100 mgs. daily was added to the regimen from week 6 to week 10. At week 10, the Evening Primrose Oil. and Pyridoxine were discontinued and the dose of Vitamin C was tapered to zero over four weeks.	16 weeks	NI	NI	NI.	One patient: subjective and objective evidence of improved oral and ocular lubrication. One patient: subjective improvement without improvement in Schirmer’s test. One patient: improvement in Schirmer’s test without subjective improvement. The others had no improvement
Pedersen et al., 1999 [38]	Denmark	40	39 F 1 M	60 (range 30–85)	The diagnosis was based on the criteria proposed by Vitali et al. (1993) [39]	NI	Three tablets of Longo Vital at breakfast. Group A: LV for the first 4 months; Group B: LV for the last 4 months.	8 months (LV: 4 months/placebo: 4 months)	Group A—Significant increase in 4 months of LV (*p* < 0.001)/Group B—no significant changes in either period	Group A—stimulated salivary flow did not change on LV but increased on the subsequent 4 months of placebo (*p* < 0.05)/Group B—stimulated salivary flow increased only on LV (*p* < 0.05)	No significant changes	NI
Peen et al., 2008 [40]	Denmark	Patients: 23; Controls: 23 (matched age and sex)	21 F 2 M	56.6 (range 34–73)	Patients fulfilling the criteria for SD proposed by the American–European Consensus Group 2002 [41]	NI	Liquid diet avoiding mastication. The patients were also offered a free fluid dietary supplement in 200 mL boxes containing 1.5 kcal/mL, including 5.6 g protein, 18.8 g carbohydrates, and 5.8 g fat per 100 mL (Fresubin Energy, Fresenius Kabi, Bad Homburg, Germany), to a maximum of 600 mL/day. Controls: not on diet.	4 weeks	Patients: week 1: 1.18 (0.73–2.03); week 4: 1.70 (0.95–2.69) *p* value: 0.02/Controls: week 1: 0.47 (0.25–0.84); week 4: 0.47 (0.02–1.05) *p* value: NS.	NI	Patients: week 1: 3.3 (0.39–4.95); week 4: 7.05 (2.39–6.55) *p* value: 0.05/Controls: week 1: 16.10 (6.64–26.31); week 4: 9.71 (4.11–12.41) *p* value: NS.	Salivary flow increased significantly in the 23 patients on the liquid diet for 4 weeks.
Singh et al., 2010 [42]	USA	61	57 F 4 M	61 (mean age)	Biopsy: NI/Focus score: NI/Anti-SSA (Ro): NI/Salivary flow: zero/Schirmer’s test: NI/Xerostomia: all patients had subjective complaints of dry mouth/Xeropthalmia: NI	All patients were on muscarinic agonists for at least 3 months.	Placebo (wheat germ oil): 23 subjects. TheraTears nutrition (*n*-3 supplement): 38 subjects. (TheraTears nutrition contains 1000 mg of flaxseed oil, 450 mg of EPA, 300 mg of DHA, 163 mg of vitamin E as d-alpha tocopherol, and 20 mg of mixed tocopherol concentrate.) One capsule per day, taken with breakfast.	3 months	*n*-3 group: UWSFR increased significantly (SD), 0.076 (0.09) mL/min at baseline to 0.140 (0.18) mL/min at 3 months *p* = 0.029. Wheat germ oil: the mean (SD) UWSFR increased slightly from 0.065 (0.08) mL/min at baseline to 0.094 (0.11) mL/min at 3 months *p* = 0.135.	*n*-3 group: 0.776 (0.74) mL/min at baseline to 1.018 (1.08) mL/min at 3 months *p* = 0.026. Wheat germ oil: the mean (SD) US increased slightly from 0.919 (0.69) at baseline to 1.02 (0.76) mL/min at 3 months *p* = 0.316.	No significant changes	Perceived improvement in dry mouth was evaluated with VAS, and it was significant in both groups.
Liao et al., 2013 [43]	Taiwan	1	M	49	Biopsy: NI/Focus score: NI/Anti-SSA (Ro): positive/Salivary flow: NI/Schirmer’s test: NI/Xerostomia: positive/Xeropthalmia: positive	Anti-La SSB: positive/antinuclear antibodies: positive (1:2560), delayed saliva excretion on salivary scintigraphy. Other symptoms: profound hypokalemia, abnormal renal function with hyperphosphaturia, and hypocalcemia.	The patient was placed on potassium citrate (45 mEq/day) and active vitamin D3 (0.25 μg/d) therapy to treat the hypokalemia and vitamin D deficiency, respectively.	NI	NI	NI	NI	The patient experienced no further sicca symptoms or paralysis during outpatient department follow-up treatment.
Goldner et al., 2024 [44]	USA	3	F	40, 54, and 45	NI	Patient 1: photosensitivity, fatigue, pain in the legs, dry skin, dry eye, dry mouth, stomach cramping, diarrhea, pelvic pain. Patient 2: photosensitivity; butterfly rash; constant fatigue; joint stiffness in the fingers, elbows, and knees; severe dry mouth and dry eye; eye inflammation; neuropathy. Patient 3: Flu-like symptoms, migraines, intermittent dizziness, weakness, dry mouth, recurrent nerve pain in skin, fatigue, light sensitivity, eye pain, trigeminal neuralgia.	Initial RRP—Include: raw vegetables (unlimited); focus on high intake of leafy greens and cruciferous vegetables, fruits, whole and ground flax and chia seeds, cold-pressed flaxseed oil, water, and vitamin B12 and D supplementation. Eliminate: all animal products, added oils, processed foods, added sugars, cooked foods, grains, and legumes. Maintenance phase—Include: vegetables (recommended 75% raw), fruits (no recommended restrictions), seeds and nuts, whole and ground flax and chia seeds, water, intact and whole grains, legumes, and vitamin B12 and D supplementation. Eliminate: all animal products, added oils, processed foods, added sugars, and alcohol.	Initial RRP: 4 weeks	NI	NI	NI	Dry mouth and eyes resolved immediately (≤1 month) after start of RRP in all 3 patients. Other symptoms also resolved with the continuation of the diet.
Al-Rawi et al., 2024 [45]	Iraq	104	99 F 5 M	Group 1: 53.4 ± 12.4; Group 2: 52.6 ± 11.3	Based on the 2016 diagnostic criteria from ACR/EULAR	NI	Group 1 was given omega-3 dietary supplements. Group 2: placebo (two capsules daily).	2 months	UWSFR at baseline (mean): Group 1: 0.99 mL/min; Group 2: 1.0 mL/min (*p* = 0.989). UWSFR at the last visit (mean): Group 1: 2.07 mL/min; Group 2: 1.55 mL/min (*p* = 0.053).	NI	Group 1: Baseline (mean): 2.38 mm; Last visit (mean): 6.63 mm (*p* < 0.001). Group 2: Baseline (mean): 2.68 mm; Last visit (mean): 5.58 mm (*p* = 0.01).	Improvements in eye symptoms including itching, mucous discharge, and photophobia. Improvement was noted in the xerostomia inventory.

Note: ACR, American College of Rheumatology; DHA, docosahexaenoic acid; EPA, eicosapentaenoic acid; EULAR, European League Against Rheumatism; F, female; GF, gluten-free; LV, Longo Vital; M, male; NI, no information; NS, not significant; RRP, rapid recovery/reversal phase; SD, Sjögren Disease; USA, United States of America; UK, United Kingdom; UWSFR, unstimulated whole salivary flow rate; VAS, visual analog scale.

## Data Availability

The original contributions presented in this study are included in the article/Supplementary Material. Further inquiries can be directed to the corresponding author.

## Supplementary Materials

The following supporting information can be downloaded at: https://www.mdpi.com/article/10.3390/nu17172743/s1.

## References

[1] Price E.J., Benjamin S., Bombardieri M., Bowman S., Carty S., Ciurtin C., Crampton B., Dawson A., Fisher B.A., Giles I. (2025). British Society for Rheumatology guideline on management of adult and juvenile onset Sjögren disease. Rheumatology.

[2] Parisis D., Chivasso C., Perret J., Soyfoo M.S., Delporte C. (2020). Current state of knowledge on primary Sjögren’s syndrome, an autoimmune exocrinopathy. J. Clin. Med..

[3] Brito-Zerón P., Baldini C., Bootsma H., Bowman S.J., Jonsson R., Mariette X., Sivils K., Theander E., Tzioufas A., Ramos-Casals M. (2016). Sjögren syndrome. Nat. Rev. Dis. Primers..

[4] Jonsson R. (2022). Disease mechanisms in Sjögren’s syndrome: What do we know?. Scand. J. Immunol..

[5] Beydon M., McCoy S., Nguyen Y., Sumida T., Mariette X., Seror R. (2024). Epidemiology of Sjögren syndrome. Nat. Rev. Rheumatol..

[6] Nocturne G., Mariette X. (2015). Sjögren Syndrome-associated lymphomas: An update on pathogenesis and management. Br. J. Haematol..

[7] Ramos-Casals M., Brito-Zerón P., Bombardieri S., Bootsma H., De Vita S., Dörner T., Fisher B.A., Gottenberg J.-E., Hernandez-Molina G., Kocher A. (2020). EULAR recommendations for the management of Sjögren’s syndrome with topical and systemic therapies. Ann. Rheum. Dis..

[8] Fox R.I., Fox C.M., Gottenberg J.E., Dörner T. (2021). Treatment of Sjögren’s syndrome: Current therapy and future directions. Rheumatology.

[9] Enger T.B., Palm Ø., Garen T., Sandvik L., Jensen J.L. (2011). Oral distress in primary Sjögren’s syndrome: Implications for health-related quality of life. Eur. J. Oral Sci..

[10] González S., Sung H., Sepúlveda D., González M., Molina C. (2014). Oral manifestations and their treatment in Sjögren’s syndrome. Oral Dis..

[11] Miyamoto S.T., Valim V., Fisher B.A. (2021). Health-related quality of life and costs in Sjögren’s syndrome. Rheumatology.

[12] Rusthen S., Young A., Herlofson B.B., Aqrawi L.A., Rykke M., Hove L.H., Palm Ø., Jensen J.L., Singh P.B. (2017). Oral disorders, saliva secretion, and oral health-related quality of life in patients with primary Sjögren’s syndrome. Eur. J. Oral Sci..

[13] Nesvold M.B., Jensen J.L., Hove L.H., Singh P.B., Young A., Palm Ø., Andersen L.F., Carlsen M.H., Iversen P.O. (2018). Dietary intake, body composition, and oral health parameters among female patients with primary Sjögren’s syndrome. Nutrients.

[14] Fernandes G., Jolly C.A. (1998). Nutrition and autoimmune disease. Nutr. Rev..

[15] Vieira S.M., Pagovich O.E., Kriegel M.A. (2014). Diet, microbiota and autoimmune diseases. Lupus.

[16] Cermak J.M., Papas A.S., Sullivan R.M., Dana M.R., Sullivan D.A. (2003). Nutrient intake in women with primary and secondary Sjögren’s syndrome. Eur. J. Clin. Nutr..

[17] Szodoray P., Horvath I.F., Papp G., Barath S., Gyimesi E., Csathy L., Kappelmayer J., Sipka S., Duttaroy A.K., Nakken B. (2010). The immunoregulatory role of vitamins A, D and E in patients with primary Sjogren’s syndrome. Rheumatology.

[18] He X., Zhao Z., Wang S., Kang J., Zhang M., Bu J., Cai X., Jia C., Li Y., Li K. (2020). High-fat diet-induced functional and pathologic changes in lacrimal gland. Am. J. Pathol..

[19] Machowicz A., Hall I., de Pablo P., Rauz S., Richards A., Higham J., Poveda-Gallego A., Imamura F., Bowman S.J., Barone F. (2020). Mediterranean diet and risk of Sjögren’s syndrome. Clin Exp Rheumatol..

[20] Zhang M., Liang Y., Liu Y., Li Y., Shen L., Shi G. (2022). High-fat diet-induced intestinal dysbiosis is associated with the exacerbation of Sjogren’s syndrome. Front. Microbiol..

[21] Haddaway N.R., Collins A.M., Coughlin D., Kirk S. (2015). The role of google scholar in evidence reviews and its applicability to grey literature searching. PLoS ONE.

[22] Moola S., Munn Z., Tufunaru C., Aromataris E., Sears K., Sfetc R., Currie M., Lisy K., Qureshi R., Mattis P., Aromataris E., Munn Z. (2020). Chapter 7: Systematic reviews of etiology and risk. JBI Manual for Evidence Synthesis.

[23] Barker T.H., Stone J.C., Sears K., Klugar M., Tufanaru C., Leonardi-Bee J., Aromataris E., Munn Z. (2023). The revised JBI critical appraisal tool for the assessment of risk of bias for randomized controlled trials. JBI Evid. Synth..

[24] Hooijmans C.R., Rovers M.M., de Vries R.B.M., Leenaars M., Ritskes-Hoitinga M., Langendam M.W. (2014). SYRCLE’s risk of bias tool for animal studies. BMC Med. Res. Methodol..

[25] Page M.J., McKenzie J.E., Bossuyt P.M., Boutron I., Hoffmann T.C., Mulrow C.D., Shamseer L., Tetzlaff J.M., Akl E.A., Brennan S.E. (2021). The PRISMA 2020 statement: An updated guideline for reporting systematic reviews. BMJ.

[26] Swanson C.A., Levy J.A., Morrow W.J. (1989). Effect of low dietary lipid on the development of Sjögren’s syndrome and haematological abnormalities in (NZB x NZW)F1 mice. Ann. Rheum. Dis..

[27] Chandrasekar B., McGuff H.S., Aufdermorte T.B., Troyer D.A., Talal N., Fernandes G. (1995). Effects of calorie restriction on transforming growth factor beta 1 and proinflammatory cytokines in murine Sjogren’s syndrome. Clin. Immunol. Immunopathol..

[28] Inoue H., Kishimoto A., Ushikoshi-Nakayama R., Hasaka A., Takahashi A., Ryo K., Muramatsu T., Ide F., Mishima K., Saito I. (2016). Resveratrol improves salivary dysfunction in a non-obese diabetic (NOD) mouse model of Sjögren’s syndrome. J. Clin. Biochem. Nutr..

[29] Guo Y., Ji W., Lu Y., Wang Y. (2021). Triptolide reduces salivary gland damage in a non-obese diabetic mice model of Sjögren’s syndrome via JAK/STAT and NF-κB signaling pathways. J. Clin. Biochem. Nutr..

[30] Haupt-Jorgensen M., Groule V., Reibel J., Buschard K., Pedersen A.M.L. (2022). Gluten-free diet modulates inflammation in salivary glands and pancreatic islets. Oral Dis..

[31] Wang Y., Xiao J., Duan Y., Miao M., Huang B., Chen J., Cheng G., Zhou X., Jin Y., He J. (2021). Lycium barbarum polysaccharide ameliorates Sjögren’s syndrome in a murine model. Mol. Nutr. Food Res..

[32] Li D., Onodera S., Deng S., Alnujaydi B., Yu Q., Zhou J. (2022). Alternate-day fasting ameliorates newly established Sjögren’s syndrome-like sialadenitis in non-obese diabetic mice. Int. J. Mol. Sci..

[33] Li D., Onodera S., Yu Q., Zhou J. (2024). The impact of alternate-day fasting on the salivary gland stem cell compartments in non-obese diabetic mice with newly established Sjögren’s syndrome. Biochim. Biophys. Acta Mol. Cell Res..

[34] Raffle R.B. (1950). Sjögren’s disease associated with a nutritional deficiency syndrome. Br. Med. J..

[35] Maclaurin B.P., Matthews N., Kilpatrick J.A. (1972). Coeliac disease associated with auto-immune thyroiditis, Sjogren’s syndrome, and a lymphocytotoxic serum factor. Aust. N. Z. J. Med..

[36] Horrobin D.F., Campbell A. (1980). Sjogren’s syndrome and the sicca syndrome: The role of prostaglandin E1 deficiency. Treatment with essential fatty acids and vitamin C. Med. Hypotheses.

[37] McKendry R.J. (1982). Treatment of Sjogren’s syndrome with essential fatty acids, pyridoxine and vitamin C. Prostaglandins Leukot. Med..

[38] Pedersen A., Gerner N., Palmvang I., Høier-Madsen M. (1999). LongoVital in the treatment of Sjögren’s syndrome. Clin. Exp. Rheumatol..

[39] Vitali C., Bombardieri S., Moutsopoulos H.M., Balestrieri G., Bencivelli W., Bernstein R.M., Bjerrum K.B., Braga S., Coll J., de Vita S. (1993). Preliminary criteria for the classification of Sjögren’s syndrome. Results of a prospective concerted action supported by the European Community. Arthritis Rheum..

[40] Peen E., Haga H.J., Haugen A.J., Kahrs G.E., Haugen M. (2008). The effect of a liquid diet on salivary flow in primary Sjögren’s syndrome. Scand. J. Rheumatol..

[41] Vitali C., Bombardieri S., Jonsson R., Moutsopoulos H.M., Alexander E.L., Carsons S.E., Daniels T.E., Fox P.C., Fox R.I., Kassan S.S. (2002). Classification criteria for Sjögren’s syndrome: A revised version of the European criteria proposed by the American-European Consensus Group. Ann. Rheum. Dis..

[42] Singh M., Stark P.C., Palmer C.A., Gilbard J.P., Papas A.S. (2010). Effect of omega-3 and vitamin E supplementation on dry mouth in patients with Sjögren’s syndrome. Spec. Care Dentist..

[43] Liao C.Y., Wang C.C., Chen I.H., Shiang J.C., Liu M.Y., Tsai M.K. (2013). Hypokalemic paralysis as a presenting manifestation of primary Sjögren’s syndrome accompanied by vitamin D deficiency. Intern. Med..

[44] Goldner B., Staffier K.L. (2024). Case series: Raw, whole, plant-based nutrition protocol rapidly reverses symptoms in three women with systemic lupus erythematosus and Sjögren’s syndrome. Front. Nutr..

[45] Al-Rawi Z.S., Jalal A.M., Hameed I.H. (2024). A randomized double-blind placebo-controlled clinical trial of fish oil (omega-3) in Sjogren’s syndrome patients in Erbil-Iraq. Mediterr. J. Rheumatol..

[46] Rhodus N.L. (1988). Qualitative nutritional intake analysis of older adults with Sjogren’s syndrome. Gerodontology.

[47] Henderson C.J., Panush R.S. (1999). Diets, dietary supplements, and nutritional therapies in rheumatic diseases. Rheum. Dis. Clin. N. Am..

[48] Semerano L., Julia C., Aitisha O., Boissier M.C. (2017). Nutrition and chronic inflammatory rheumatic disease. Jt. Bone Spine.

[49] Nobs S.P., Zmora N., Elinav E. (2020). Nutrition regulates innate immunity in health and disease. Annu. Rev. Nutr..

[50] Schwager J., Seifert N., Bompard A., Raederstorff D., Bendik I. (2021). Resveratrol, EGCG and vitamins modulate activated T lymphocytes. Molecules.

[51] Liu Q. (2011). Triptolide and its expanding multiple pharmacological functions. Int. Immunopharmacol..

[52] Moudgil K.D., Venkatesha S.H. (2022). The anti-inflammatory and immunomodulatory activities of natural products to control autoimmune inflammation. Int. J. Mol. Sci..

[53] Chien K.J., Horng C.T., Huang Y.S., Hsieh Y.H., Wang C.J., Yang J.S., Lu C., Chen F. (2018). Effects of Lycium barbarum (goji berry) on dry eye disease in rats. Mol. Med. Rep..

[54] Chang K., Luo P., Guo Z., Yang L., Pu J., Han F., Cai F., Tang J., Wang X. (2025). Lipid metabolism: An emerging player in Sjögren’s syndrome. Clin. Rev. Allergy Immunol..

[55] Mora J.R., Iwata M., von Andrian U.H. (2008). Vitamin effects on the immune system: Vitamins A and D take centre stage. Nat. Rev. Immunol..

[56] Radić M., Kolak E., Đogaš H., Gelemanović A., Nenadić D.B., Vučković M., Radić J. (2023). Vitamin D and Sjögren’s disease: Revealing the connections-a systematic review and meta-analysis. Nutrients.

[57] Isola S., Gammeri L., Furci F., Gangemi S., Pioggia G., Allegra A. (2024). Vitamin C supplementation in the treatment of autoimmune and onco-hematological diseases: From prophylaxis to adjuvant therapy. Int. J. Mol. Sci..

[58] Simopoulos A.P. (2002). Omega-3 fatty acids in inflammation and autoimmune diseases. J. Am. Coll. Nutr..

[59] Poggioli R., Hirani K., Jogani V.G., Ricordi C. (2023). Modulation of inflammation and immunity by omega-3 fatty acids: A possible role for prevention and to halt disease progression in autoimmune, viral, and age-related disorders. Eur. Rev. Med. Pharmacol. Sci..

[60] Castrejón-Morales C.Y., Granados-Portillo O., Cruz-Bautista I., Ruiz-Quintero N., Manjarrez I., Lima G., Hernández-Ramírez D.F., Astudillo-Angel M., Llorente L., Hernández-Molina G. (2020). Omega-3 and omega-6 fatty acids in primary Sjögren’s syndrome: Clinical meaning and association with inflammation. Clin. Exp. Rheumatol..

[61] da Nave C.B., Pereira P., Silva M.L. (2024). The effect of polyunsaturated fatty acid (PUFA) supplementation on clinical manifestations and inflammatory parameters in individuals with Sjögren’s syndrome: A literature review of randomized controlled clinical trials. Nutrients.

[62] Maarse F., Jager D.H.J., Alterch S., Korfage A., Forouzanfar T., Vissink A., Brand H.S. (2019). Sjögren’s syndrome is not a risk factor for periodontal disease: A systematic review. Clin. Exp. Rheumatol..

[63] Lerner A., Shoenfeld Y., Matthias T. (2017). Adverse effects of gluten ingestion and advantages of gluten withdrawal in nonceliac autoimmune disease. Nutr. Rev..

[64] Bruzzese V., Scolieri P., Pepe J. (2021). Efficacy of gluten-free diet in patients with rheumatoid arthritis. Reumatismo.

[65] Lerner A., de Carvalho J.F., Kotrova A., Shoenfeld Y. (2022). Gluten-free diet can ameliorate the symptoms of non-celiac autoimmune diseases. Nutr. Rev..

[66] Clemente-Suárez V.J., Beltrán-Velasco A.I., Redondo-Flórez L., Martín-Rodríguez A., Tornero-Aguilera J.F. (2023). Global impacts of Western diet and its effects on metabolism and health: A narrative review. Nutrients.

[67] Manzel A., Muller D.N., Hafler D.A., Erdman S.E., Linker R.A., Kleinewietfeld M. (2014). Role of “Western diet” in inflammatory autoimmune diseases. Curr. Allergy Asthma Rep..

[68] Malesza I.J., Malesza M., Walkowiak J., Mussin N., Walkowiak D., Aringazina R., Bartkowiak-Wieczorek J., Mądry E. (2021). High-fat, Western-style diet, systemic inflammation, and gut microbiota: A narrative review. Cells.

[69] Deng C., Xiao Q., Fei Y. (2022). A glimpse into the microbiome of Sjögren’s syndrome. Front. Immunol..

[70] de Carvalho J.F., Lerner A., Gonçalves C.M., Shoenfeld Y. (2021). Sjögren syndrome associated with protein-losing enteropathy: Case-based review. Clin. Rheumatol..

[71] Medina G., Vera-Lastra O., Peralta-Amaro A.L., Jiménez-Arellano M.P., Saavedra M.A., Cruz-Domínguez M.P., Jara L.J. (2018). Metabolic syndrome, autoimmunity and rheumatic diseases. Pharmacol. Res..

[72] Augusto K.L., Bonfa E., Pereira R.M.R., Bueno C., Leon E.P., Viana V.S.T., Pasoto S.G. (2016). Metabolic syndrome in Sjögren’s syndrome patients: A relevant concern for clinical monitoring. Clin. Rheumatol..

[73] Servioli L., Maciel G., Nannini C., Crowson C.S., Matteson E.L., Cornec D., Berti A. (2019). Association of smoking and obesity on the risk of developing primary Sjögren syndrome: A population-based cohort study. J. Rheumatol..

[74] Carubbi F., Alunno A., Mai F., Mercuri A., Centorame D., Cipollone J., Mariani F.M., Rossi M., Bartoloni E., Grassi D. (2021). Adherence to the Mediterranean diet and the impact on clinical features in primary Sjögren’s syndrome. Clin. Exp. Rheumatol..

[75] Xiao Y.-L., Gong Y., Qi Y.-J., Shao Z.-M., Jiang Y.-Z. (2024). Effects of dietary intervention on human diseases: Molecular mechanisms and therapeutic potential. Signal Transduct. Target. Ther..

[76] Alwarith J., Kahleova H., Rembert E., Yonas W., Dort S., Calcagno M., Burgess N., Crosby L., Barnard N.D. (2019). Nutrition interventions in rheumatoid arthritis: The potential use of plant-based diets. A review. Front. Nutr..

[77] Knippenberg A., Robinson G.A., Wincup C., Ciurtin C., Jury E.C., Kalea A.Z. (2022). Plant-based dietary changes may improve symptoms in patients with systemic lupus erythematosus. Lupus.

[78] Chaaya C., Raad E., Kahale F., Chelala E., Ziade N., Maalouly G. (2025). Adherence to Mediterranean diet and ocular dryness severity in Sjögren’s syndrome: A cross-sectional study. Med. Sci..

[79] Pringle S., Wang X., Verstappen G.M.P.J., Terpstra J.H., Zhang C.K., He A., Patel V., Jones R.E., Baird D.M., Spijkervet F.K.L. (2019). Salivary gland stem cells age prematurely in primary Sjögren’s syndrome. Arthritis Rheumatol..

[80] Li R.-N., Ou T.-T., Lin C.-H., Lin Y.-Z., Fang T.-J., Chen Y.-J., Tseng C.-C., Sung W.-Y., Wu C.-C., Yen J.-H. (2023). NLRP3 gene polymorphisms in rheumatoid arthritis and primary Sjogren’s syndrome patients. Diagnostics.

[81] He J., Xu M., Chen Y., Wu S. (2024). Grp78 regulates NLRP3 inflammasome and participates in Sjogren’s syndrome. Int. Immunopharmacol..

[82] Duregon E., Pomatto-Watson L.C.D., Bernier M., Price N.L., de Cabo R. (2021). Intermittent fasting: From calories to time restriction. Geroscience.

[83] Okawa T., Nagai M., Hase K. (2021). Dietary intervention impacts immune cell functions and dynamics by inducing metabolic rewiring. Front. Immunol..

[84] Procaccini C., de Candia P., Russo C., De Rosa G., Lepore M.T., Colamatteo A., Matarese G. (2024). Caloric restriction for the immunometabolic control of human health. Cardiovasc. Res..

[85] Humphreys-Beher M.G., Brayer J., Yamachika S., Peck A.B., Jonsson R. (1999). An alternative perspective to the immune response in autoimmune exocrinopathy: Induction of functional quiescence rather than destructive autoaggression. Scand. J. Immunol..

[86] Hall H.D., Schneyer C.A. (1964). Salivary gland atrophy in rat induced by liquid diet. Proc. Soc. Exp. Biol. Med..

[87] Takahashi S., Nezu A., Tanimura A., Nakamichi Y., Yamamoto T. (2022). Responses of salivary glands to intake of soft diet. J. Oral Biosci..

[88] Shiboski C.H., Shiboski S.C., Seror R., Criswell L.A., Labetoulle M., Lietman T.M., Rasmussen A., Scofield H., Vitali C., Bowman S.J. (2017). 2016 American College of Rheumatology/European League Against Rheumatism Classification Criteria for primary Sjögren’s syndrome: A consensus and data-driven methodology involving three international patient cohorts. Arthritis Rheumatol..

[89] Nagi R., Kumar S.S., Sheth M., Deshpande A., Khan J. (2025). Association between oral microbiome dysbiosis and Sjogren Syndrome. A systematic review of clinical studies. Arch. Oral Biol..

[90] Bellando-Randone S., Russo E., Venerito V., Matucci-Cerinic M., Iannone F., Tangaro S., Amedei A. (2021). Exploring the Oral Microbiome in Rheumatic Diseases, State of Art and Future Prospective in Personalized Medicine with an AI Approach. J. Pers. Med..

[91] Alam J., Lee A., Lee J., Kwon D.I., Park H.K., Park J.-H., Jeon S., Baek K., Lee J., Park S.-H. (2020). Dysbiotic oral microbiota and infected salivary glands in Sjögren’s syndrome. PLoS ONE.

[92] Tseng Y.-C., Liao K.-S., Lin W.-T., Li C., Chang C.-B., Hsu J.-W., Chan C.-P., Chen C.-M., Wang H.-P., Chien H.-C. (2025). A human oral commensal-mediated protection against Sjögren’s syndrome with maintenance of T cell immune homeostasis and improved oral microbiota. NPJ Biofilms Microbiomes.

[93] Richards J.L., Yap Y.-A., McLeod K.H., Mackay C.R., Mariño E. (2016). Dietary metabolites and the gut microbiota: An alternative approach to control inflammatory and autoimmune diseases. Clin. Transl. Immunol..

[94] Osailan S.M., Pramanik R., Shirlaw P., Proctor G.B., Challacombe S.J. (2012). Clinical assessment of oral dryness: Development of a scoring system related to salivary flow and mucosal wetness. Oral Surg. Oral Med. Oral Pathol. Oral Radiol..

