# Supplementary material for: Nutritional Intervention for Sjögren Disease: A Systematic Review
Source: Nutrients. 2025 Aug 25;17(17):2743. doi: 10.3390/nu17172743 (PMC12430580; doi:10.3390/nu17172743)
Supplement: Supplementary file 1 [file nutrients-17-02743-s001.zip › nutrients-3803301-supplementary.pdf]

## Supplementary files

# Nutritional intervention for Sjögren disease: a systematic review

## Table of contents

**Supplementary file S1.** Search strategies employed to identify articles in electronic databases……………………………………………………………..………….**2**

**Supplementary file S2.** Joanna Briggs Institute (JBI) critical appraisal checklist for case reports……………………………………………………………………….…..…….**3**

**Supplementary file S3.** Joanna Briggs Institute (JBI) critical appraisal checklist for case-control studies…………………..…………………………………………………..……**4**

**Supplementary file S4.** Joanna Briggs Institute (JBI) critical appraisal checklist for Randomized Controlled Trials…………………………………………………….………..**5**

**Supplementary file S5.** Supplementary file 5. SYRCLE Risk of Bias tool for animal studies ………………………………………………………………………..…………….**6**

**Supplementary file S1.** Search strategies employed to identify articles in electronic databases

| | |
|---|---|
| PubMed | (("Sjogren's Syndrome" OR "Sjogrens Syndrome" OR "Syndrome, Sjogren's" OR "Sjogren Syndrome" OR "Sicca Syndrome" OR "Syndrome, Sicca" OR Sjögren or Sjogren) AND (nutrition OR "nutritional status" OR "Status, Nutritional" OR "Nutrition Status" OR "Status, Nutrition" OR nutritionist OR diet OR diets OR nutrient OR nutrients OR Malnutrition OR "vitamin supplementation" OR "oral supplementation" OR "calorie restriction" OR "dietary intervention" OR "dietary patterns")) AND (xerostomia OR Xerostomias OR Hyposalivation OR Hyposalivations OR "dry mouth" OR "salivary gland") |
| Embase | ('sjogrens syndrome' OR 'sjogren syndrome' OR 'sicca syndrome' OR 'syndrome, sicca' OR sjögren OR sjogren) AND (nutrition OR 'nutritional status' OR 'status, nutritional' OR 'nutrition status' OR 'status, nutrition' OR nutritionist OR diet OR diets OR nutrient OR nutrients OR malnutrition OR 'vitamin supplementation' OR 'oral supplementation' OR 'calorie restriction' OR 'dietary intervention' OR 'dietary patterns') AND (xerostomia OR xerostomias OR hyposalivation OR hyposalivations OR 'dry mouth' OR 'salivary gland') |
| Web of Science and Scopus | ((ALL=(xerostomia OR xerostomia OR Hyposalivation OR hyposalivation OR "dry mouth" OR "salivary gland")) AND ALL=(nutrition OR “nutritional status” OR “Status, Nutritional” OR “Nutrition Status” OR “Status, Nutrition” OR nutritionist OR diet OR diets OR nutrient OR nutrients OR Malnutrition OR "vitamin supplementation" OR "oral supplementation" OR "calorie restriction" OR "dietary intervention" OR "dietary patterns")) AND ALL=("Sjogren's Syndrome" OR "Sjogrens Syndrome" OR "Syndrome, Sjogren's" OR "Sjogren Syndrome" OR "Sicca Syndrome" OR "Syndrome, Sicca" OR Sjögren or Sjogren) |

**Supplementary file S2.** Joanna Briggs Institute (JBI) critical appraisal checklist for case reports

| Study | Were the patient's demographic characteristics clearly described? | Was the patient's history clearly described and presented as a timeline? | Was the current clinical condition of the patient on presentation clearly described? | Were diagnostic tests or asses0073ment methods and the results clearly described? | Was the intervention(s) or treatment procedure(s) clearly described? | Was the post-intervention clinical condition clearly described? | Were adverse events (harms) or unanticipated events identified and described? | Does the case report provide takeaway lessons? |
|---|---|---|---|---|---|---|---|---|
| Raffle, 1950 | Yes | Yes | Yes | Yes | Yes | No | No | Yes |
| Maclaurin et al., 1972 | Yes | Yes | Yes | Yes | Yes | Yes | No | Yes |
| Horrobin, 1980 | Yes | Yes | No | No | No | Yes | No | Yes |
| McKendry, 1982 | No | No | No | Yes | Yes | Yes | Unclear | Yes |
| Liao et al., 2013 | Yes | Yes | Yes | Yes | Yes | Yes | No | Yes |
| Goldner et al., 2024 | Yes | Yes | Yes | Yes | Yes | Yes | No | Yes |

**Supplementary file S3.** Joanna Briggs Institute (JBI) critical appraisal checklist for case-control studies

| Study | Were the groups comparable other than the presence of disease in cases or the absence of disease in controls? | Were cases and controls matched appropriately? | Were the same criteria used for identification of cases and controls? | Was exposure measured in a standard, valid and reliable way? | Was exposure measured in the same way for cases and controls? | Were confounding factors identified? | Were strategies to deal with confounding factors stated? | Were outcomes assessed in a standard, valid and reliable way for cases and controls? | Was the exposure period of interest long enough to be meaningful? | Was appropriate statistical analysis used? |
|---|---|---|---|---|---|---|---|---|---|---|
| Peen et al., 2008 | Yes | Yes | Unclear | Yes | Yes | Yes | No | Unclear | Unclear | Yes |

**Supplementary file S4.** Joanna Briggs Institute (JBI) critical appraisal checklist for Randomized Controlled Trials

| Study | Was true randomization used for assignment of participants to treatment groups? | Was allocation to treatment groups concealed? | Were treatment groups similar at the baseline? | Were participants blind to treatment assignment? | Were those delivering the treatment blind to treatment assignment? | Were treatment groups treated identically other than the intervention of interest? | Were outcome assessors blind to treatment assignment? | Were outcomes measured in the same way for treatment groups? | Were outcomes measured in a reliable way | Was follow up complete and if not, were differences between groups in terms of their follow up adequately described and analysed? | Were participants analysed in the groups to which they were randomized? | Was appropriate statistical analysis used? | Was the trial design appropriate and any deviations from the standard RCT design (individual randomization, parallel groups) accounted for in the conduct and analysis of the trial? |
|---|---|---|---|---|---|---|---|---|---|---|---|---|---|
| Al-Rawi et al., 2024 | Yes | Yes | Yes | Yes | Yes | Yes | Yes | Yes | Yes | Yes | Yes | Unclear | Yes |
| Singh et al., 2010 | Unclear | No | Yes | Yes | Yes | Yes | Unclear | Yes | Unclear | Yes | Yes | Unclear | Yes |
| Pedersen et al., 1999 | Unclear | Unclear | Yes | Yes | Yes | Yes | Unclear | Yes | Yes | Yes | Yes | Yes | Yes |

**Supplementary file S5.** SYRCLE Risk of Bias tool for animal studies

| Study | Was the allocation sequence adequately generated and applied? | Were the groups similar at baseline or were they adjusted for confounders in the analysis? | Was the allocation adequately concealed? | Were the animals randomly housed during the experiment? | Were the caregivers and /or investigators blinded from knowledge which intervention each animal received during the experiment | Were animals selected at random for outcome assessment? | Was the outcome assessor blinded? | Were incomplete outcome data adequately addressed? | Are reports of the study free of selective outcome reporting? | Was the study apparently free of other problems that could result in high risk of bias? |
|---|---|---|---|---|---|---|---|---|---|---|
| Swanson et al., 1989 | Unclear | Yes | No | No | No | Unclear | Yes | Unclear | Yes | No |
| Chandrasekar et al., 1995 | Unclear | Yes | No | No | No | Unclear | No | Unclear | Yes | No |
| Inoue et al., 2016 | Unclear | Yes | Unclear | No | No | Unclear | No | Unclear | Yes | No |
| Guo et al., 2021 | Unclear | Yes | No | Unclear | No | Unclear | No | Unclear | Unclear | No |
| Wang et al., 2021 | Unclear | Yes | Unclear | Yes | No | Yes | No | Unclear | Yes | No |
| Haupt-Jorgensen et al., 2021 | Unclear | Yes | Unclear | Unclear | No | Yes | Yes | Yes | Yes | No |
| Zhang et al., 2022 | Unclear | Yes | Unclear | Yes | No | Unclear | Yes | Unclear | Yes | No |
| Li et al., 2022 | Unclear | Yes | No | No | No | Unclear | No | Unclear | Yes | No |
| Li et al., 2024 | Unclear | Yes | Unclear | Unclear | No | Unclear | No | Unclear | Yes | No |
